# A database of ocean primary productivity from the ^14^C method

**DOI:** 10.1002/lol2.10175

**Published:** 2020-11-16

**Authors:** J. F. Marra, R. T. Barber, E. Barber, R. R. Bidigare, W. S. Chamberlin, R. Goericke, B. R. Hargreaves, M. Hiscock, R. Iturriaga, Z. I. Johnson, D. A. Kiefer, C. Kinkade, C. Knudson, V. Lance, C. Langdon, Z.-P. Lee, M. J. Perry, W. O. Smith, R. Vaillancourt, L. Zoffoli

**Affiliations:** 1 Brooklyn College, The City University of New York, New York City, New York; 2 Duke University, Durham, North Carolina; 3 University of Hawaii at Manoa, Honolulu, Hawaii; 4 Fullerton College, Fullerton, California; 5 Scripps Institution of Oceanography, San Diego, California; 6 Lehigh University, Bethlehem, Pennsylvania; 7 Environmental Protection Agency, Washington, DC; 8 University of Southern California, Los Angeles, California; 9 National Oceanographic and Atmospheric Administration, Woods Hole, MA; 10 Lamont-Doherty Earth Observatory, Columbia University, Palisades, New York; 11 University of Maryland, College Park, Maryland; 12 Rosenstiel School of Marine and Atmospheric Sciences, University of Miami, Miami, Florida; 13 University of Massachusetts Boston, Boston, Massachusetts; 14 University of Maine, Orono, Maine; 15 Virginia Institute of Marine Sciences, College of William and Mary, Gloucester Point, Virginia; 16 Millersville University, Millersville, Pennsylvania; 17 University of Nantes, Nantes, France

## Abstract

The database on ocean primary productivity comprises over two decades (1985–2008) of data that the authors have participated in collecting, using the assimilation of inorganic ^14^C through photosynthesis, in incubations carried out in situ. The dataset is perhaps unique in that it uses, overwhelmingly, consistent methodology while covering a wide geographic range. Ancillary data are included. Using the database, it is hoped that investigators can test for the relationships among the environmental drivers for ocean productivity, the meaning of the ^14^C method in terms of phytoplankton physiology and the dynamics in the water column, and as a resource for further development of productivity algorithms using satellite ocean color imagery.

In 1952, E. Steemann Nielsen published his “^14^C technique” (Steemann Nielsen 1952) to measure C assimilation as a proxy of net primary production in aquatic systems. He submitted a first manuscript while conducting measurements aboard the *Galathea* expedition (1950–1952), to illustrate its efficacy, and thereby introduced a new means to understand ocean productivity. Compared to oxygen flux measurements used in the 1930s and 1940s, the ^14^C method, as it came to be known, had unparalleled sensitivity and ease of use. Nevertheless, almost from the beginning, the method aroused controversy (initiated by Steemann Nielsen himself). By the 1970s, however, the method attained wide enough use that maps of global ocean productivity were being produced (Koblentz-Mishke et al. 1970), albeit with little independent validation.

The advantage of the ^14^C method for measuring photosynthetic carbon assimilation in the ocean is its extreme sensitivity. Earlier methods, notably, the analysis of oxygen changes in incubated samples, simply cannot discriminate the small changes in O_2_ characteristic of many regions of the ocean. The second advantage is the ^14^C method’s relative facility. It requires (in addition to the isotope) only a means to separate the particulate matter from the seawater, and a means to assay the radioactivity. Although it is an unstable isotope, handling ^14^C in the activities used is safe, requiring no special equipment. At sea, precautions for all isotopes must be taken to ensure the ship itself does not become contaminated.

One of the corollaries to the extreme sensitivity to the ^14^C method is that, early on, it could not be validated or compared with other measurements. Perhaps that, and the ease with which the measurements could be made, is why it took so long to recognize significant concerns (*see* Marra 2002). By the late 1970s, criticisms were being made regarding the effects of incubation, respiration, the activities of heterotrophs, and other concerns. Some of these issues persist to this day. It had become clear that the biochemistry of the ^14^C method was not completely understood. Its most important advantage, sensitivity to low rates of production, became a liability in that, as noted above, it could not be compared to other measures. However, research programs in the 1980s (Eppley 1982) put the ^14^C method on firmer foundation. Additional methodological concerns, for example, so-called bottle effects, are discussed in Marra (2009).

The ^14^C method for measuring primary productivity was a core measurement in the Joint Global Ocean Flux (JGOFS) program in the 1990s. It was employed along with other methods to understand imbalances in the ocean’s carbon cycle from seasonal monsoons, equatorial upwelling, and seasonality in the North Atlantic and Southern Oceans (Hanson et al., 2000). JGOFS not only established international protocols (Knap et al. 1996), used later in other programs, it also produced a body of data based on a consistent method over a wide range of oceanic conditions.

The era of satellite ocean color began in earnest, with the launches of the Coastal Zone Color Scanner (CZCS) (1978–1986) and the Sea-Viewing Wide Field-of-View Sensor (SeaWiFS) (1997–2010). Satellite sensing of near-surface ocean color provided the opportunity to estimate productivity from space over the global ocean at unprecedented scales of time and space; the ^14^C method became the means for translating estimates of biomass into a rate of primary production. Various “productivity algorithms” were soon tested using the results from primary productivity based on the ^14^C method (Campbell et al. 2002; Carr et al. 2006).

Here, we present a new compendium of primary productivity data based on ^14^C. This database differs from others that we are aware (*see*, e.g., Buitenhuis et al. 2013) in that we confine the measurements to incubations done in situ (not in deck incubators), and to using a consistent methodology. In addition, we include as much ancillary data as possible. Ancillary variables include pigments, phytoplankton absorption, irradiance values, nutrients, temperatures, etc. The database has wide geographic coverage and extends over 20 years (1985–2008). The database will be useful to understand global ocean productivity in terms of physical, chemical, and other biological variables, and to help in improving algorithms for understanding the magnitude and variability of ocean productivity estimated from ocean color satellites. Despite its limitations, the ^14^C method remains the preeminent technique for measuring oceanic productivity at sea.

## Methods

The procedure for conducting the in situ experiments generally followed protocols prescribed by JGOFS (Knap et al. 1996). While JGOFS protocols call for 24-h incubations (and results from these are what were reported to the JGOFS database), we often did contemporaneous 12-h (dawn-dusk) incubations. Pigments were assessed using fluorometry (Turner Designs) and also using high-performance liquid chromatography (HPLC) following the methods outlined in Trees et al. (2000) and Van Heukelem and Thomas (2001). Water temperatures and nutrient concentrations were provided by various support projects and collaborators, and followed standard methods. Methodological details for the data from JGOFS can be found at the U.S. JGOFS website (http://usjgofs.whoi.edu/), in Barber et al. (2001) and Marra et al. (1995).

We make note regarding some of the variables. Mixed layer depth (MLD) is available for most of the JGOFS data, and is defined as the density difference from the surface to the depth of an increase in density of 0.03 g cm^−3^ (Gardner et al. 1993). MLDs using other criteria are available at the JGOFS website (http://usjgofs.whoi.edu/). There are two types of incubation containers used, tissue culture flasks and polycarbonate bottles. For JGOFS data, the depth of sampling can vary slightly from the depth of incubation. We have listed both (“Z_sample” and “Z_incubation”) for completeness. Again, for completeness, we have listed names for “sites” where they exist, and which are names assigned to various station locations.

Overviews of the various multi-instiutional programs used in the database are as follows: PRPOOS (Plankton Rate Processes in Oligotrophic Oceans; Eppley 1982), Biowatt (Marra and Hartwig 1984), Marine Light-Mixed Layers (Marra 1988, Marra 1995), and JGOFS (Brewer et al., 1986). Kinkade et al. (1997) has background for Arlindo.

A few publications have used the ancillary data in analyses of productivity. Those interested in the use of pigment data (for example) can consult Marra et al. (1993) and Marra et al. (2000). The latter publication describes methods for pigment and spectral absorption (e.g., the “filterpad technique”). Marra (2009) reviews some of the earlier publications arising from the data.

## Results

The geographic coverage of the database is shown in Fig. 1. Table 1 shows the programs that produced the data, and keyed to the identifiers in Fig. 1. Example results from the database shown in Fig. 2 are data from two cruises to the Arabian Sea. These are by no means the limit of what can be accessed or analyzed.

Figure 2 illustrates, and as we have found for other programs, that photoinhibition, a markedly lower rate of carbon assimilation near the ocean surface, is not usually observed. A lower value for carbon assimilation near the surface is often a feature in simulated in situ incubations, those carried out in deck incubators (Barber et al. 1997, 2001). The difference might be explained by the difference in irradiance exposure between the two methods (the deck incubators were blue plexiglass to compensate for spectral changes). Unlike incubations on deck, near-surface samples incubated in situ will experience less irradiance because of a higher sun angle (lower zenith angle) near sunrise and sunset meaning greater reflection and less irradiance entering the surface layers. The samples incubated on deck are always exposed to direct solar irradiance, and therefore may experience photo-inhibiting levels of irradiance to a greater degree compared to those incubated in situ. Deeper samples are light-limited and are expected to have the opposite response, with deck-incubated samples having higher assimilation than their in situ counterparts. The competing effects may produce the often-observed comparable values for depth-integrated primary production between the two methods (*see* Barber et al. 2001). Another factor is the imperfect matching of changes to spectral irradiance in the deck incubators. (See Barber et al. (1997) for a discussion and a method to correct for differences.)

Related with the aforementioned, in an initial analysis of the data, the depth profile of chlorophyll *a* is, generally, independent of the depth profile of carbon assimilation (Fig. 2, right panel).

## Discussion

There are two other databases that we are aware of, one held at Oregon State University (OSU) and another at University of East Anglia (reported in Buitenhuis et al. 2013). The OSU database, in its earliest compilation, was used in the development of the VGPM algorithm (Behrenfeld and Falkowski 1997). At that time, a major contributor to the database was the Marine Resources Monitoring, Assessment and Prediction program on the continental shelf of the northeastern U.S. The OSU database now has data from the Equatorial Pacific (contributed by R. T. Barber). In addition to time and location, the OSU database has irradiance data at light depths. The Hawaii Ocean Time-Series (hahana.soest.hawaii.edu/hot/) and the Bermuda Atlantic Time Series (bats.bios.edu) also maintain data that are accessible on-line and consist of in situ carbon assimilation over depth for incubations lasting from dawn to dusk.

In the two other databases described above, however, it is not always clear how the incubations were done, that is, whether on-deck or in situ, and accompanying environmental information is limited. We chose to include here as much supporting and ancillary data as possible, with the idea that including them might lead to the emergence of new predictive relationships.

## Figures and Tables

**Fig 1.**
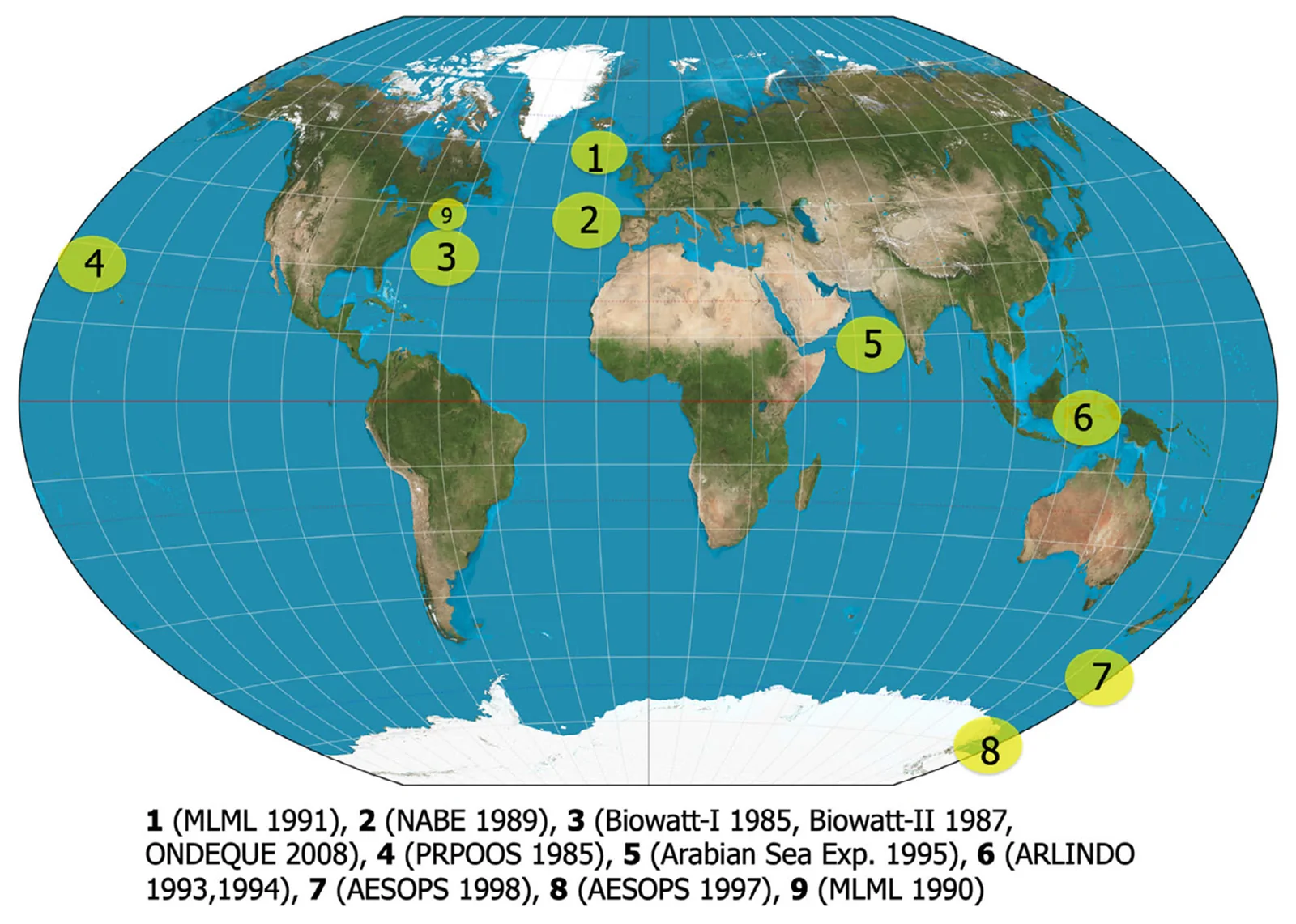
Locations where primary productivity experiments were done. Further details are in Table 1.

**Fig 2.**
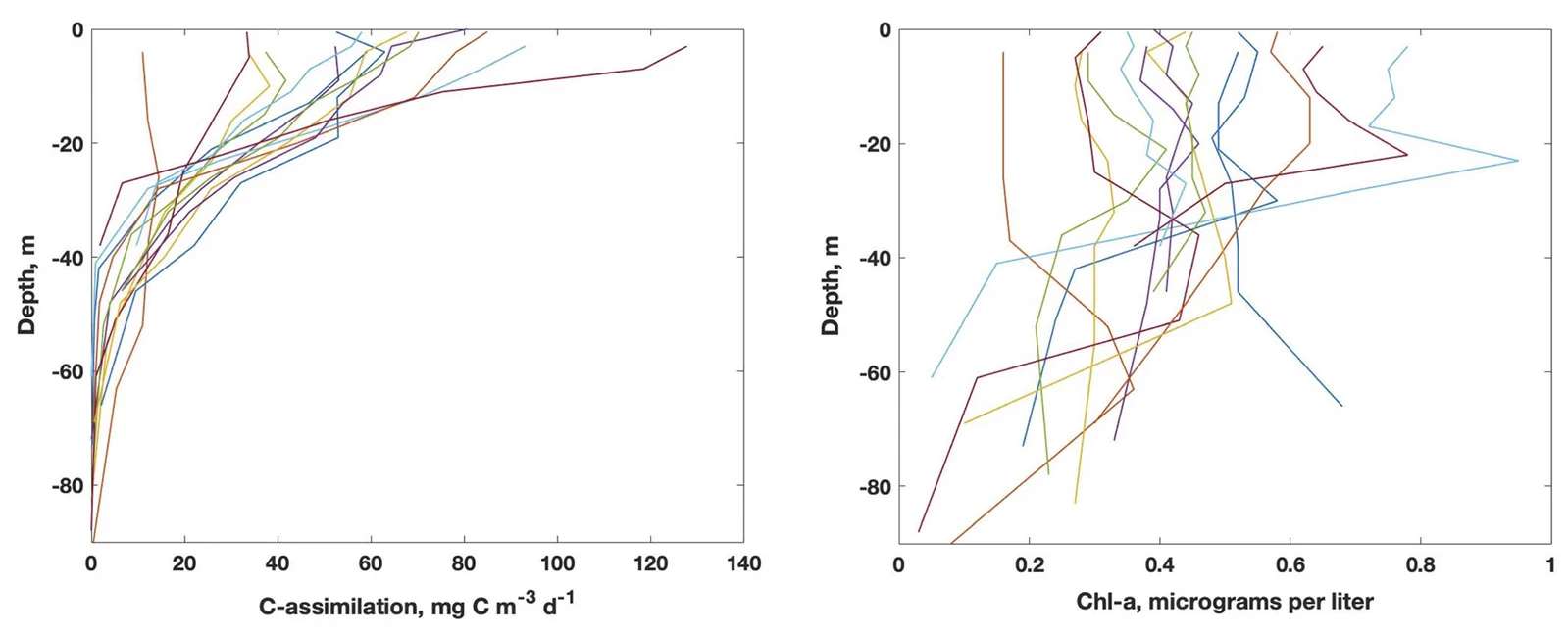
Examples of data on carbon assimilation and chlorophyll *a* as a function of depth using data from the Arabian Sea Expedition, northeast and southwest monsoons (*see* Barber et al., 2001).

**Table 1.** Data available.

Program, location	Map no.	Year	No. of productivity profiles
PRPOOS’85, N. Pac. Central Gyre	4	1985	4
Biowatt1 Biowatt2, N. Sargasso Sea	3	1985, 1987	20
North Atlantic Bloom Experiment	2	1989	12
ML-ML, Gulf of Maine	9	1990	4
ML-ML, Iceland Basin	1	1991	7
Arlindo, Indonesian Seas	6	1993–1994	10
Arabian Sea Expedition	5	1995	42
AESOPS, Ross Sea	8	1997	12
AESOPS, Polar Front	7	1997–1998	15
On Deque, N. Sargasso Sea	3	2008	7

## Data Availability

The In Situ Primary Productivity dataset is freely available at the Biological and Chemical Oceanography Data Management Office (BCO-DMO) (bco-dmo.org) at http://dmoserv3.bco-dmo.org/jg/serv/BCO-DMO/ISPP14C/c14_primary_prod.html0%7Bdir=dmoserv3.whoi.edu/jg/dir/BCO-DMO/ON_DEQUE3/,info=dmoserv3.bco-dmo.org/jg/info/BCO-DMO/ISPP14C/c14_primary_prod%7D . The data are arrayed in a text file (“Flat Listing”), but can be downloaded in other formats. The rows are each data record, and the columns denote the variables. The DOI issued by BCO-DMO is 10.26008/1912/bco-dmo.814803.1.

## Scientific Significance Statement

A database of oceanic primary productivity is described. The database is freely available. The database is from observations using consistent methodology, and has wide geographic coverage. The database will prove useful in understanding the environmental drivers of primary productivity in the ocean, and as a resource for the development of algorithms for estimating ocean productivity from satellite sensors.
